# Intestinal Microbiota at Engraftment Influence Acute Graft-Versus-Host Disease *via* the Treg/Th17 Balance in Allo-HSCT Recipients

**DOI:** 10.3389/fimmu.2018.00669

**Published:** 2018-04-24

**Authors:** Lijie Han, Hua Jin, Lizhi Zhou, Xin Zhang, Zhiping Fan, Min Dai, Qianyun Lin, Fen Huang, Li Xuan, Haiyan Zhang, Qifa Liu

**Affiliations:** ^1^Department of Hematology, Nanfang Hospital, Southern Medical University, Guangzhou, China; ^2^Department of Biostatistics, Southern Medical University, Guangzhou, China; ^3^Department of Gastroenterology, Nanfang Hospital, Southern Medical University, Guangzhou, China

**Keywords:** intestinal microbiota, histone acetylation, immune homeostasis, acute graft-versus-host disease, allogeneic hematopoietic stem cell transplantation

## Abstract

Animal models have indicated that intestinal microbiota influence acute graft-versus-host disease (aGVHD) by modulating immune homeostasis. But, in humans, the mechanism by which the microbiota induces aGVHD remains unclear. In this study, we investigated the relationship between the intestinal microbiota and T cell subsets in patients who undergo allogeneic hematopoietic stem cell transplantation (allo-HSCT) to explore the mechanism by which microbiota induced aGVHD. Based on aGVHD, this study was categorized into two groups: grades II–IV aGVHD (aGVHD group, *n* = 32) and grade 0–I aGVHD (non-aGVHD group, *n* = 49). The intestinal microbiota was detected by 16S rRNA gene sequencing, and the T cell subsets and histone 3 (H3) acetylation in CD4+ T cells in the peripheral blood was assayed by flow cytometry at the time of engraftment. The aGVHD group had greater low microbial diversity than the non-aGVHD group (56.3 versus 24.5%, *p* = 0.004). The bacterial community was depleted of Clostridia (e.g., the Lachnospiraceae and Ruminococcaceae families) and enriched for Gammaproteobacteria (e.g., the Enterobacteriaceae family) in the aGVHD group compared with the non-aGVHD group. The relative abundance of Lachnospiraceae and Ruminococcaceae was positively correlated with the Treg/Th17 ratio counts (*r* = 0.469 and 0.419; *p* < 0.001 and <0.001, respectively), whereas Enterobacteriaceae was negatively correlated with the Treg/Th17 ratio (*r* = −0.277; *p* = 0.012). The level of acetylated H3 in CD4+ T cells was not only correlated with Lachnospiraceae/Ruminococcaceae, but also with the Treg/Th17 ratio (*r* = 0.354; *p* = 0.001). In conclusions, our results suggest that decreased Lachnospiraceae and Ruminococcaceae and increased Enterobacteriaceae, correlate with a Treg/Th17 imbalance, which might be through acetylated H3 in CD4+ T cells. These findings suggest that intestinal microbiota might induce aGVHD by influencing the Treg/Th17 balance.

## Introduction

Allogeneic hematopoietic stem cell transplantation (allo-HSCT) is a potentially curative treatment for a variety of hematologic malignancies. Major causes for limiting allo-HSCT include some life-threatening complications, such as graft-versus-host disease (GVHD) and infections (1–4). GVHD is categorized as acute GVHD (aGVHD) and chronic GVHD according to the time of occurrence and clinical manifestations. A variety of factors impact GVHD, such as HLA type, donor source, donor or patient age, and conditioning regimen (5–9). In addition, emerging evidences have demonstrated that the intestinal microbiome plays a vital role in the morbidity and mortality of acute graft-versus-host disease (aGVHD) during allo-HSCT, and loss of microbiota diversity can promote the development of aGVHD (7, 10, 11).

Loss of diversity, including expansion of Enterobacteriales and loss of Clostridiales, was observed in animal models of GVHD (12, 13). Other experiments have demonstrated that a decrease in intestinal microbiota-derived short-chain fatty acids (SCFAs) in intestinal epithelial cells is associated with GVHD after allo-HSCT, whereas supplementation with Clostridia, which highly produce SCFAs, could mitigate GVHD (14). In humans, a previous study has suggested Enterococcus increases before or at the onset of aGVHD (15). In contrast, recent studies have indicated that at the time of engraftment decreased Clostridia, such as Lachnospiraceae and Bifidobacteriaceae, are associated with subsequent aGVHD post-transplantation (13, 16). With regards to the mechanism of these microbiota members in aGVHD, animal experiments have suggested that Clostridia metabolites could mitigate intestinal barrier damage and induce immune tolerance (14, 17, 18), which depend on the levels of SCFAs and histone acetylation that is influenced by SCFAs (17, 19–21). In addition, pathogenic bacteria disrupt immune tolerance by activating T cells, and alterations in the indigenous microbiota disrupt immune homeostasis, resulting in the development of aGVHD (20, 22–24). However, in humans, particularly in patients prior to aGVHD, the mechanism underlying the specific microbiota taxa and immune homeostasis remains unclear.

To explore the mechanism of the effects of the intestinal microbiota on immune homeostasis prior to aGVHD in patients post-transplantation, we performed a retrospective analysis of a prospective study of recipients undergoing allo-HSCT. The results demonstrate that the relative abundance of Lachnospiraceae, Ruminococcaceae, and Enterobacteriaceae correlated with the Treg/Th17 balance. Additionally, the level of histone 3 (H3) acetylation in CD4 T cells not only correlated with Lachnospiraceae/Ruminococcaceae, but it also correlated with the Treg/Th17 ratio. These findings suggest that the composition of the intestinal microbiome might be determined by regulating the level of acetylated H3 in CD4 T cells and influence the development of aGVHD *via* the Treg/Th17 balance during allo-HSCT.

## Materials and Methods

### Samples

Samples were collected from adult patients who underwent allo-HSCT in our institution. Feces samples were collected at the time of engraftment and stored in a biospecimen bank. Blood samples were directly examined by flow cytometry. For each subject, biospecimens were collected within 72 h of stem cell engraftment (absolute neutrophil count: 0.5*10^7^/L for three consecutive days) after allo-HSCT.

### Conditioning and GVHD Prophylaxis

As previously described (25), conditioning regimens included standard myeloablative and intensified conditioning regimens. Standard conditioning included total body irradiation (TBI) + cyclophosphamide (Cy) and busulfan (Bu) + Cy. Intensified conditioning included 30 mg/m^2^/day fludarabine (Flu) and 2 g/m^2^/day cytarabine (on days −10 to −6), 4.5 Gy TBI/day (on days −5 and −4), and 60 mg/kg/day cyclophosphamide (Cy) and 600 mg/day etoposide (on days −3 and −2).

Cyclosporin A (CsA) and methotrexate (MTX) (on days +1, + 3, +6) were administered to patients who underwent matched sibling donor transplants for GVHD prophylaxis. CsA + MTX + ATG (ATG, Thymoglobulin; Genzyme, Cambridge) + mycophenolate (MMF) was administered to patients who underwent haploidentical donor transplants for GVHD (26).

### Infection Prophylaxis

At our institution, antimicrobial prophylaxis was routinely administered to patients as previously described (26, 27). Oral sulfamethoxazole and norfloxacin were used in all cases for infection prophylaxis. Our institution does not administer antibiotics, such as metronidazole, for the purpose of gut decontamination and/or GVHD prevention. Patients were given carbapenem (or a combination with amikacin) as a standard antibiotic for fever during neutropenia. Acyclovir and ganciclovir were given for the prophylaxis and treatment of cytomegalovirus infection. Antifungal agents were used for fungal infection prophylaxis. Fluconazole (0.3 g/day) or itraconazole (0.4 g/kg/day) was administered up to 60 days post-transplantation in patients with no history of invasive fungal infection (IFI); those with a history of IFI received voriconazole (0.4 g/day), itraconazole (0.4 g/day), caspofungin (50 mg/day), or ambisome (2 mg/kg/day) intravenously. Oral voriconazole or itraconazole was used when the peripheral white blood cell count was greater than 2.0 × 10^9^/L and was discontinued after 90 days post-transplantation.

### DNA Extraction and 16S rRNA Gene Sequencing for Fecal Specimens

For each fecal specimen, DNA was extracted and purified, and the V3–V4 region of the 16S rRNA genes was polymerase chain reaction (PCR)-amplified using modified universal bacterial primers (16). Purified PCR products were sequenced with the Hiseq2500 PE250 platform (28). Sequence data were compiled and processed using mothur version 1.31.2. Sequence data were screened and filtered for quality and then aligned to the full-length 16S rRNA gene, using the SILVA reference alignment as a template. Sequences were grouped into operational taxonomic units (OTUs) of 97% similarity (29).

### Microbial Diversity Analysis

A non-parametric test (Mann–Whitney) was used to compare the statistical significance between the II–IV aGVHD and non-aGVHD groups. OTU-based alpha diversity was analyzed by calculating observed OTUs, the Shannon index using QIIME workflow (11, 30). Microbial diversity was also estimated by calculating the inverse Simpson index, an ecological estimate of diversity calculated to represent the reciprocal of the expected probability of randomly selected bacterial sequences belonging to the same OTU. Microbial diversity was divided into the groups high (≥2) and low (<2) based on the inverse Simpson index at post-engraftment time (7, 11). Phylogenetic classification at the family and genus levels was analyzed based on the naive Bayesian classification scheme and Greengenes reference database (30).

### Linear Discriminant Analysis (LDA) Effect Size (LEfSe) Analysis

To identify the different microbiota taxa between the II–IV aGVHD and non-aGVHD groups. LEfSe analysis was performed using LEfSe software. The non-parametric factorial Kruskal–Wallis rank-sum test was used in LEfSe analysis to detect characteristics that are significantly different in abundance between the II–IV aGVHD and non-aGVHD groups. The effect sizes of the identified characteristics were then analyzed with LDA model (31).

### Detection of T Lymphocyte Subsets and Acetylated Histones in CD4+ T Cells in Peripheral Blood

Intracellular staining was examined using the Intracellular Staining Kit (BD Pharmingen, San Diego, CA, USA). Cells were incubated for 5 h with phorbol-12-myristate-13-acetate (50 ng/ml) plus ionomycin (2.5 µg/ml, all reagents from Sigma Chemical) to stimulate IL-17A production. The sample was supplemented with Golgistop (0.7 µl/ml) during the last 4 h to trap the proteins in the cytoplasm. T lymphocyte subsets [CD45+, CD3+, CD4+, CD25+, and Foxp3+ (i.e., Treg cells) and CD45+, CD3+, CD4+, CD8−, and IL-17A+ (i.e., Th17 cells)] in peripheral blood were examined by flow cytometry. The percentage of Treg and Th17 cells was classified as the percentage of Treg and Th17 cells in the CD4+ T cells population. Acetylated H3 and H4 in CD4+ CD8− T cells were also detected by flow cytometry and analyzed according to their median fluorescence intensity (MFI) (32).

### Statistical Analysis

Analysis was performed on June 29, 2017. The data are summarized as the mean ± SD or median for continuous data. Comparisons of categorical variables were performed using Pearson’s χ^2^ test or Fisher’s exact test when appropriate. The Cox proportional hazards regression model was used to analyze risk factors for aGVHD. Factors that were associated with aGVHD with *p*-values less than 0.10 in univariate analysis or other factors, such as age, donor type, vancomycin, that may influence aGVHD were included in the final models. Statistical correlations between the microbiota and T lymphocyte subsets and acetylated histone after transplantation were investigated using bivariate correlation analysis (Spearman). All *p*-values were two-sided with the significance level fixed at 0.05. SPSS 19.0 (SPSS, Chicago) or R software (version 3.1.1) were used for all analyses. *p* < 0.05 indicated statistically significant differences.

## Results

### Patient, Donor, and Transplant Characteristics

Between January 2015 and December 2016, from an original cohort of 99 patients, 18 were excluded either because the patients died before engraftment (*n* = 1), or there were disqualifying issues with the fecal or blood specimens collected (*n* = 17). In total, 81 patients were included in the statistical analysis, the cases were categorized into two groups: grades II–IV aGVHD (aGVHD group, *n* = 32) including 13 cases with grades III–IV aGVHD, and grade 0–I aGVHD (non-aGVHD group, *n* = 49). Of the 32 patients with grades II–IV aGVHD, 27 patients had ≥2 target tissue involved and 26 cases had intestinal symptom. The patient, donor, and transplant characteristics are summarized in Table 1. Most of the transplant characteristics of the two groups are similar, including donor type, hematopoietic-cell transplantation comorbidity index (HCT-CI), underlying disease, disease status, donor source, and TBI conditioning regimen. However, the aGVHD group had more cases that received b-lactam antibiotics, and underwent intensified conditioning compared with the non-aGVHD group (*p* = 0.006 and 0.049, respectively, Table 1).

**Table 1:** Patient and transplant characteristics.

Variable	Non-acute graft-versus-host disease (aGVHD) (*n* = 49)	II–IV aGVHD (*n* = 32)	*p*
Age, median years (range) <33 (%)	33 (16–54) 22 (44.9)	33 (17–55) 15 (46.9)	0.869 0.861
**Gender (%)**			
Female	19 (38.8)	11 (34.4)	0.688
**Donor gender (%)**
Female	13 (26.5)	7 (21.9)	0.635
**Pretransplant comorbidity [Hematopoietic-cell transplantation comorbidity index (HCT-CI)]**			
0–1 2–3	41 (83.4) 8 (16.3)	23 (71.9) 9 (28.1)	0.202
**Underlying disease (%)**			
AML ALL MDS	29 (59.2) 17 (34.7) 3 (6.1)	20 (62.5) 10 (31.3) 2 (6.2)	0.949
**Disease risk**			
High (%) Low or intermediate	26 (53.1) 23 (46.9)	19 (59.4) 13 (40.6)	0.576
**Disease status**			
CR Non-CR	39 (79.6) 10 (20.4)	25 (78.1) 7 (21.9)	0.874
**Conditioning (%)**			
Intensified Standard	10 (20.4) 39 (79.6)	13 (40.6) 19 (59.4)	0.049
TBI Non-TBI	17 (34.7) 32 (65.3)	8 (25.0) 24 (75.0)	0.356
**Donor type (%)**			
MSD HID	26 (53.1) 23 (46.9)	15 (46.9) 17 (53.1)	0.653
**Graft source, No. (%)**			
BM + PBSC PBSC	19 (38.8) 30 (61.2)	11 (34.4) 21 (65.6)	0.688
Cell yield MNC (median) (10^8^/kg)	8.1(4.3–14.6)	8.2 (5.4–14.3)	0.587
**Antibiotics (%)^^a^^**			
b-lactam^b^ Vancomycin (intravenous) Amikacin	36 (73.5) 25 (51.0) 24 (49.0)	31 (96.9) 20 (62.5) 19 (59.4)	0.006 0.309 0.359
Bloodstream infection	8 (16.3)	8 (25.0)	0.338

*^a^Evaluated between conditioning and engraftment time*.

*^b^b-lactams include carbapenem, cephalosporin, and b-lactam-b-lactamase combinations*.

*ALL, acute lymphoblastic leukemia; AML, acute myelogenous leukemia; BM, bone marrow; HID, haploidentical donor; MDS, myelodysplastic syndrome; MNC, mononuclear cell; MSD, matched sibling donor; PBSC, peripheral blood stem cell; TBI, total body irradiation*.

### AGVHD-Associated Intestinal Microbiota Characteristics at Engraftment

**Figure 1:**
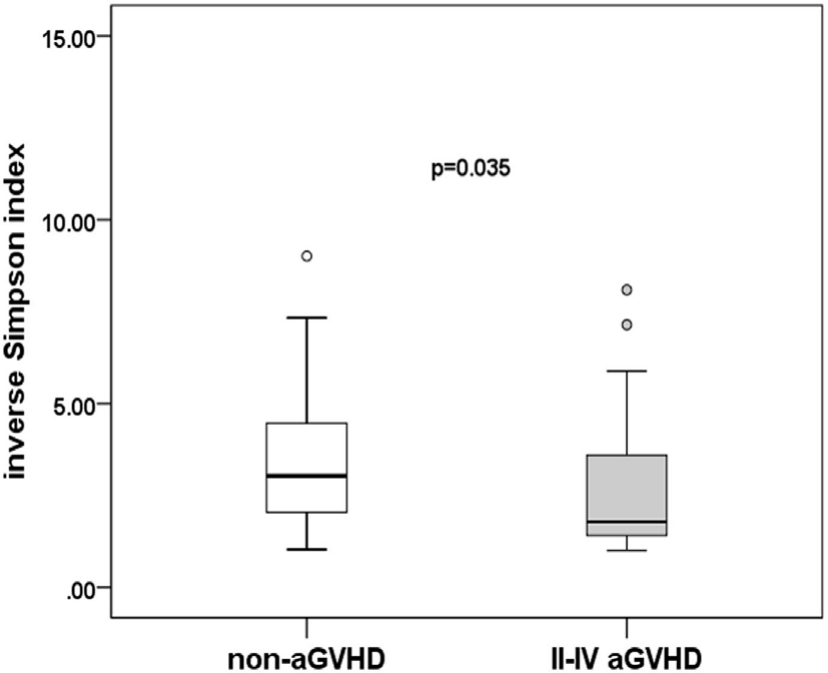
Difference of diversity in the intestinal microbiota. The intestinal microbiota diversity at engraftment is expressed as the inverse Simpson index according to the groups.

**Figure 2:**
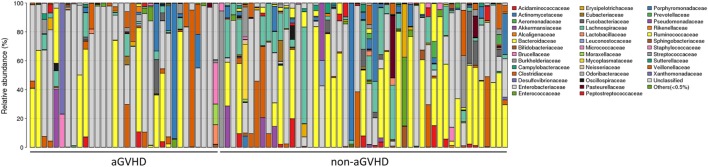
The intestinal ecosystem at engraftment between acute graft-versus-host disease (aGVHD) and non-aGVHD patients. Each rank is a study subject, which represents the phylogenetic composition of each subject. The relative abundance of intestinal bacterial taxa is shown.

To assess differences in intestinal microbiota between the aGVHD and non-aGVHD groups, we investigated the diversity and richness of the ecosystem between the groups at the time of engraftment. Alpha diversity, as measured by the inverse Simpson index, was lower in the aGVHD group (1.78, range: 1.00–8.09) compared with the non-aGVHD group (3.03, range: 1.02–9.01; *p* = 0.035; Figure 1). Consistently, estimations with observed OTUs and the Shannon index were also demonstrated to be significantly lower in the aGVHD group compared with the non-aGVHD group (*p* < 0.01 and <0.01; Figures S1A,B in Supplementary Material). The richness of the microbiota in the fecal samples is shown in Figure 2. These results indicate that the taxonomic composition of the fecal microbiota may be less complex in the aGVHD group with fewer distinct members. In subjects with aGVHD, the microbiome was generally dominated by a single microbiota family.

**Figure 3:**
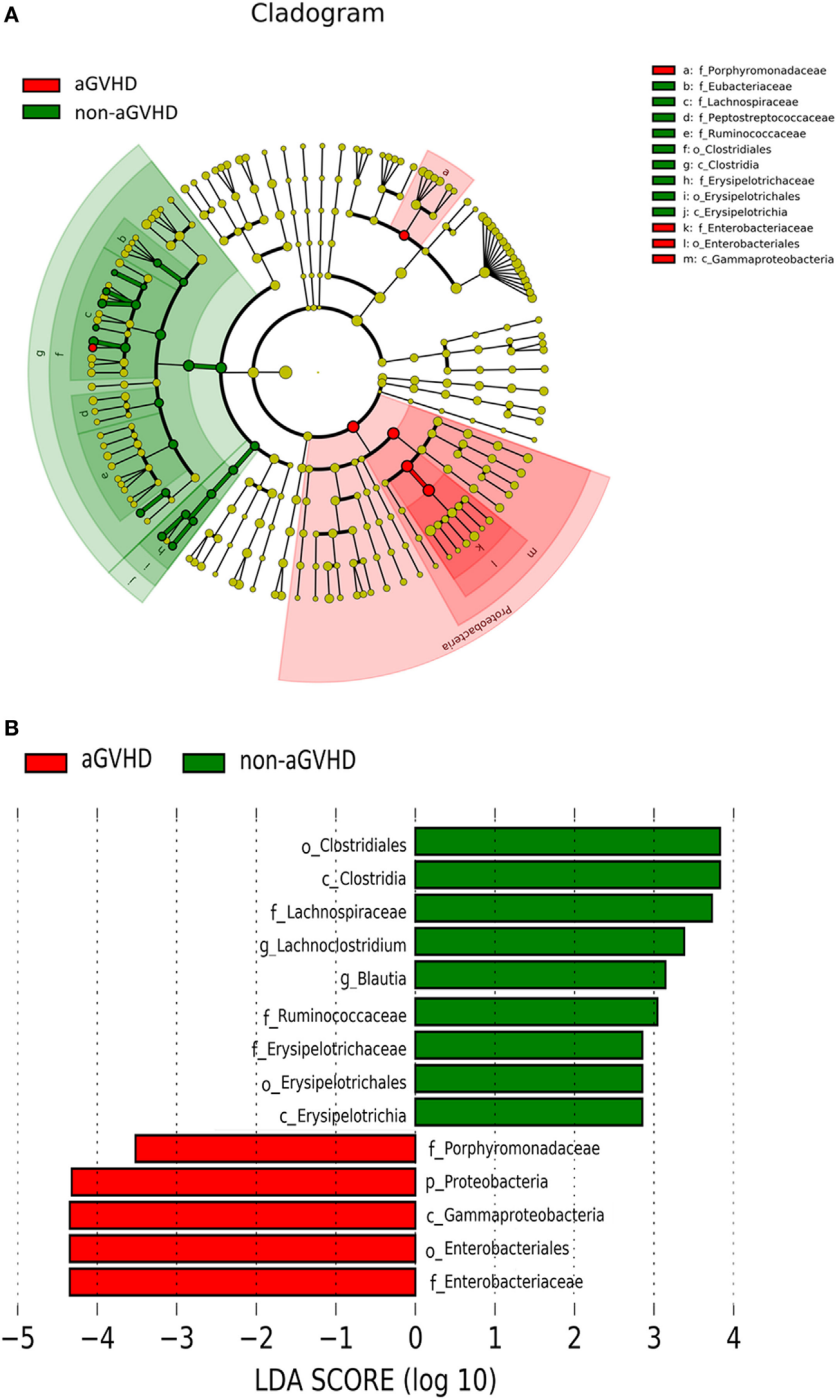
Differences of intestinal microbiota at engraftment between acute graft-versus-host disease (aGVHD) and non-aGVHD patients. **(A)** The taxonomic cladogram was generated using linear discriminate analysis (LDA) effect size (LEfSe) analysis between the groups. All listed microbiota groups are significantly different (*p* < 0.05, Kruskal–Wallis test). **(B)** The histogram shown is an additional representation of analysis by the LEfSe method. All listed microbiota groups are significantly different (*p* < 0.05, Kruskal–Wallis test). Abbreviations: p, phylum; c, class; o, order; f, family; g, genus.

To determine whether the absence or presence of a specific bacterial taxa was associated with aGVHD, we compared the microbiota composition of the patients in the two groups (Figures 3A,B). The post-engraftment microbiota of the aGVHD group differed significantly from the non-aGVHD group. At the phylum level, the microbiota community in the aGVHD group was enriched for Proteobacteria compared with the non-aGVHD group (*p* = 0.021, Figures 3A,B), and specific class and family in Proteobacteria, including Gammaproteobacteria and Enterobacteriaceae, were higher in the aGVHD group compared with the non-aGVHD group (*p* = 0.023, 0.023, respectively, Figures 3A,B). Although the relative abundance of the phyla Firmicutes was not significantly different between the two groups, a specific class of Firmicutes (Clostridia) was lower in the aGVHD group compared with the non-aGVHD group (*p* = 0.006, Figures 3A,B), and its families i.e., Lachnospiraceae and Ruminococcaceae, were also significantly less in the aGVHD group compared with the non-aGVHD group (*p* = 0.001 and 0.007, respectively, Figures 3A,B). We also identified other families’ commensal bacteria, e.g., Peptostreptococcaceae, which were significantly depleted in the aGVHD group, but abundant in the non-GVHD group (*p* = 0.004, Figure 3A). At the genus level, the relative abundance of Blautia and *Lachnoclostridium* in the aGVHD group was also significantly lower than that in the non-aGVHD group (*p* = 0.004 and 0.001, respectively, Figure 3B).

### Risk Factors for aGVHD

According to the Cox regression model for multivariate analysis of aGVHD shown in Table 2, b-lactam antibiotics administration and low microbial diversity were independent risk factors for aGVHD [*p* = 0.041 and 0.024; hazard ratio (HR) = 4.485 (1.060–18.985) and 2.386 (1.120–5.083), respectively]. Intensified conditioning was not an independent risk factor for aGVHD although it was associated with aGVHD in univariate analysis. However, patient age, donor type, vancomycin (intravenous) therapy were not identified as being associated with aGVHD (*p* > 0.05).

**Table 2:** Multivariate analysis of acute graft-versus-host disease (aGVHD).

Factors	II–IV aGVHD	
	Univariate [*p* (HR)]	Multivariate [*p* (HR, 95% CI)]
Patient age		
≤32 years >32 years	0.866 (0.942)	0.493 (1.299, 0.615–2.742)
Donor type		
MSD HID	0.447 (1.309)	0.302 (1.521, 0.686–3.373)
Conditioning		
Intensified Standard	0.042 (2.083)	0.849 (1.086, 0.462–2.556)
b-lactam^a^	0.037 (8.338, 1.137–61.118)	0.039 (8.407, 1.118–63.237)
Vancomycin (intravenous)	0.317 (1.441)	0.584 (1.228, 0.589–2.557)
Microbial diversity High^b^ (inverse Simpson ≥2) Low (inverse Simpson <2)	0.005 (2.759)	0.029 (2.415, 1.092–5.340)

*^a^b-lactams, include carbapenem, cephalosporin, and b-lactam-b-lactamase combinations*.

*^b^Measured by inverse Simpson index: high diversity ≥2; low diversity <2*.

*CI, confidence interval; HID, haploidentical donor; HR, hazard ratio; MSD, matched sibling donor*.

### Association Between Patient, Transplant Characteristics, and Intestinal Microbiota

**Figure 4:**
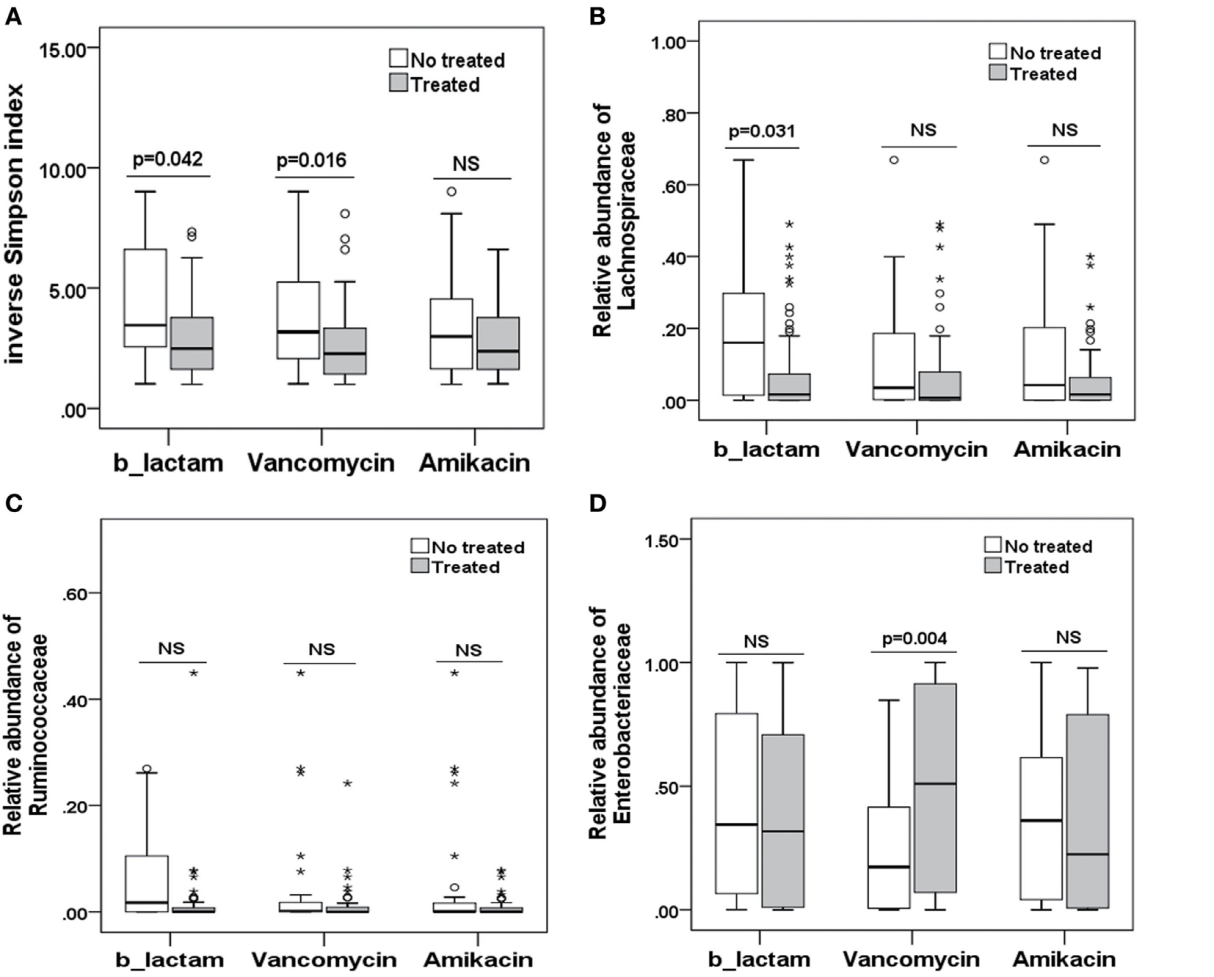
Differences in the intestinal microbiota (diversity and composition) are associated with special antibiotic administration in allo-HSCT patients. **(A)** Differences in microbiota diversity, **(B)** relative abundance of the Lachnospiraceae, **(C)** Ruminococcaceae, and **(D)** Enterobacteriaceae families under different antibiotics. b-lactams, include carbapenems, cephalosporins, and b-lactam-b-lactamase combinations.

**Figure 5:**
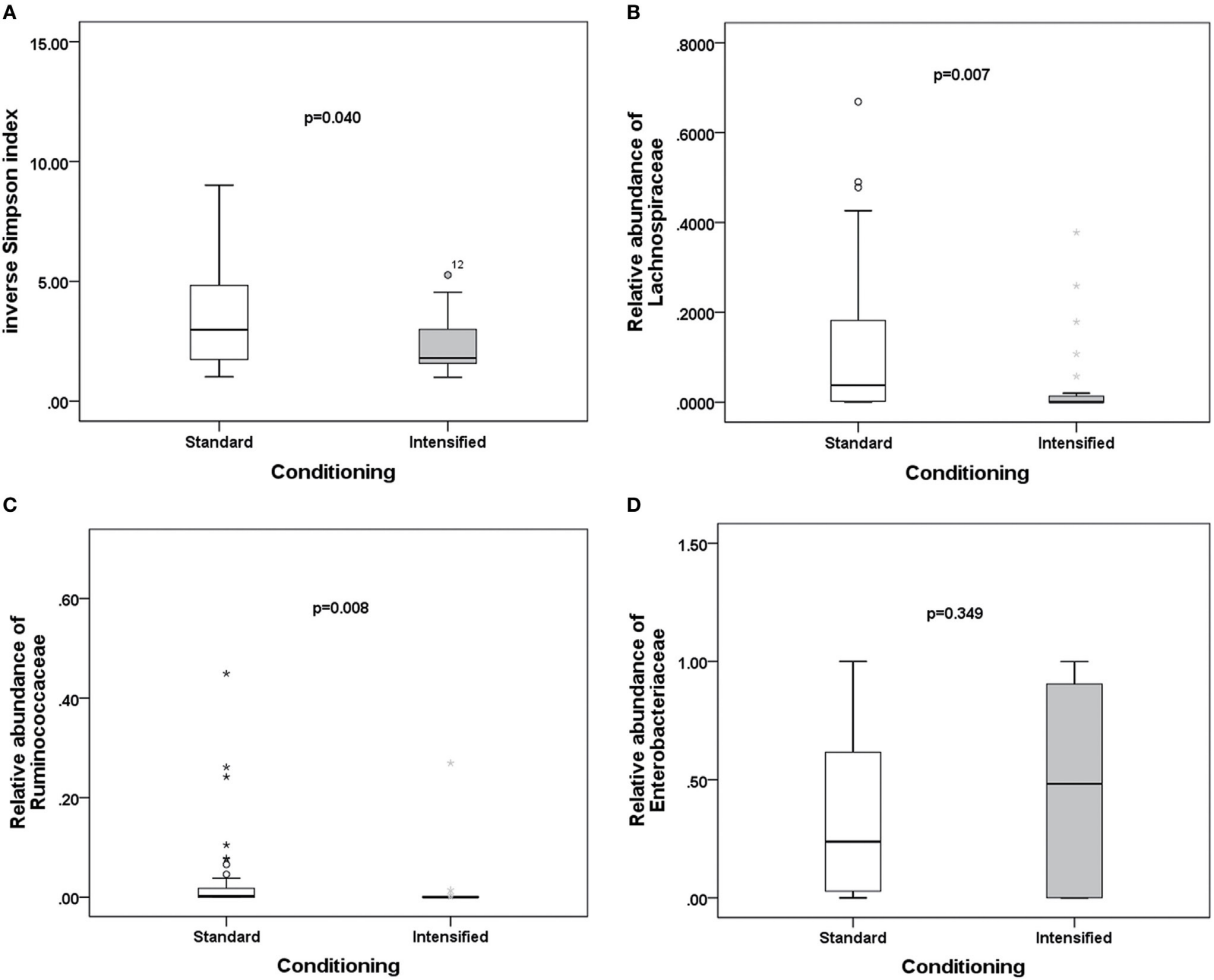
Differences in the intestinal microbiota (diversity and composition) are associated with conditioning intensity in allo-HSCT patients. **(A)** Differences in microbiota diversity, **(B)** relative abundance of the Lachnospiraceae, **(C)** Ruminococcaceae, and **(D)** Enterobacteriaceae families between standard and intensified conditioning.

To explore potential factors influencing the intestinal microbiota, we investigated the patient, donor, and transplant characteristics (Table 1). We first analyzed the effects of antibiotics on the microbiota. A total of 79 patients received antibiotics for neutropenic fever before engraftment, and two patients were not administered antibiotics. Carbapenem (or its combination with an amikacin), which belongs to b-lactam family, such as imipenem/cilastatin, was the first-line antibiotic for empiric fever and neutropenia. Vancomycin or piperacillin/tazobactam was used as second-line antibiotics. Administration of other antibiotics was variable, and these were used for a minority of patients. For instance, tigecycline was occasionally used for non-effective second-line antibiotics for bacterial infections, and fluoroquinolone was used for slight infections. These results indicate that microbial diversity is influenced by both b-lactam and vancomycin (*p* = 0.042 and 0.007, respectively; Figure 4A). However, b-lactam resulted in significant loss of the Lachnospiraceae family other than Ruminococcaceae (*p* = 0.031 and 0.089, respectively; Figures 4B,C), whereas vancomycin resulted in a bloom of the Enterobacteriaceae family (*p* = 0.002; Figure 4D). Amikacin was not found to have significant effects on the microbiota. Second, we found that intensified conditioning also had an effect on microbial diversity (*p* = 0.040; Figure 5A), resulting in significant loss of the Lachnospiraceae and Ruminococcaceae families (*p* = 0.007 and 0.008, respectively; Figures 5B,C), although Enterobacteriaceae was not influenced significantly (*p* = 0.349, Figure 5D). Additionally, we found no other factors associated with the microbiota, including patient age, sex, HCT-CI, underlying disease, disease status, donor type and source, TBI conditioning regimen, or bloodstream infection (*p* > 0.05).

### The Intestinal Microbiota Correlates With the Balance of Treg and Th17 Cells

**Figure 6:**
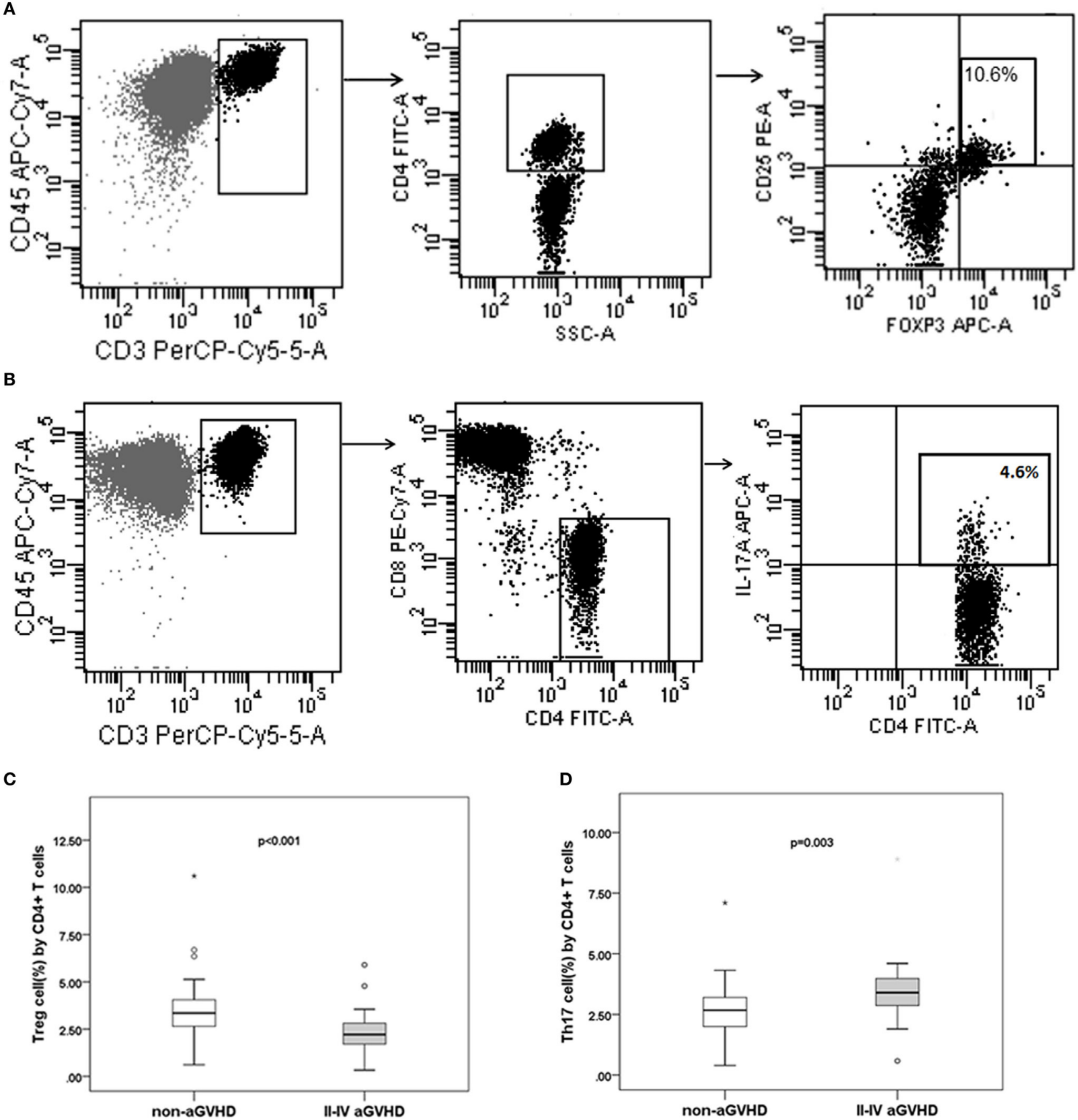

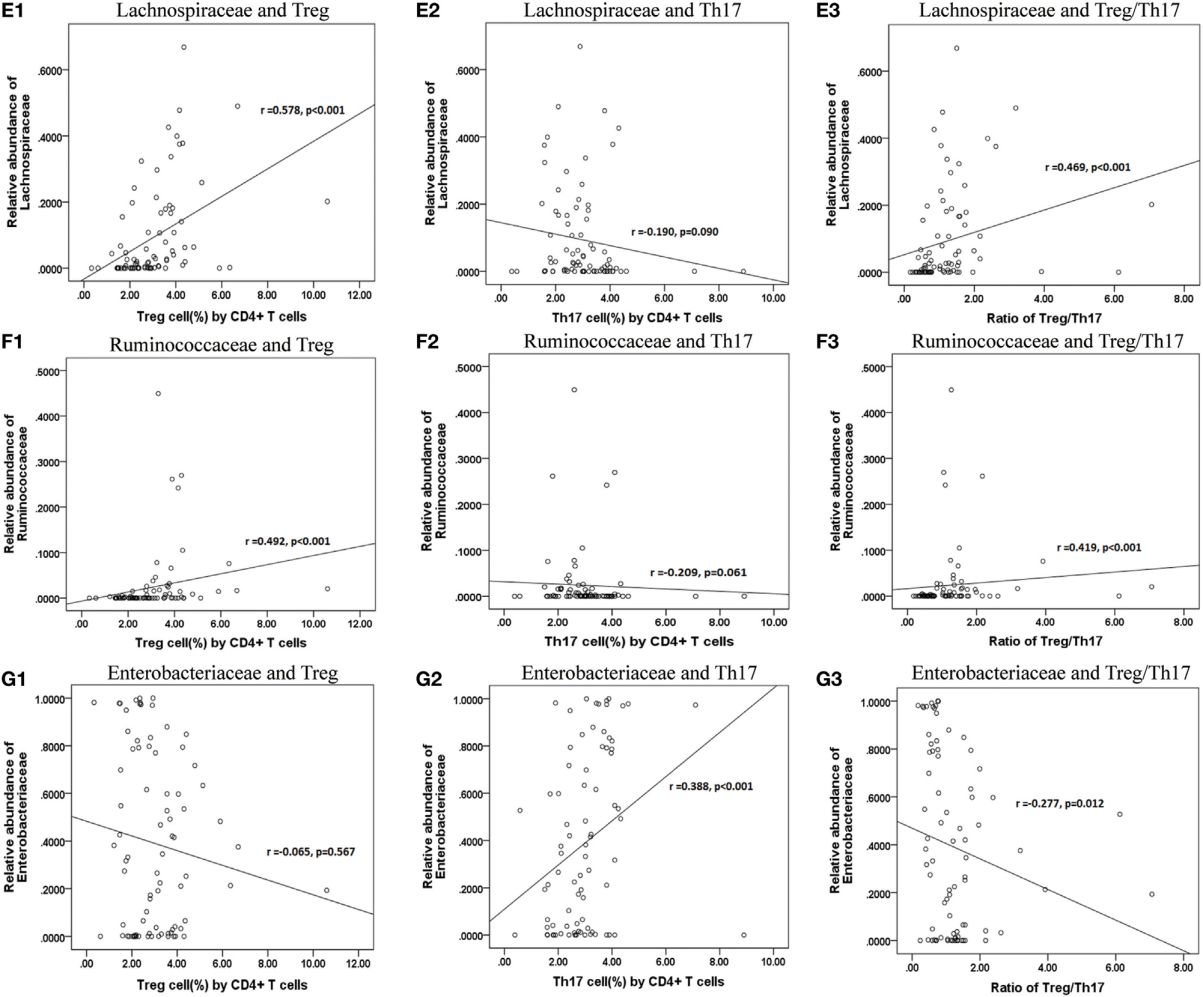
The percentage of Treg and Th17 cell counts are associated with aGVHD and intestinal microbiota correlates with the Treg/Th17 balance. The phenotype of regular T cells (Treg) **(A)** and IL-17-producing T cells Th17 **(B)** in peripheral mononuclear blood cells was examined. The percentage of Treg **(C)** and Th17 **(D)** cells is shown for the two groups. Correlations between the relative abundance of microbiota and percentage of T subsets and the Treg/Th17 ratio (**E1**: Lachnospiraceae and Treg; **E2**: Lachnospiraceae and Th17; **E3**: Lachnospiraceae and Treg/Th17. **F1**: Ruminococcaceae and Treg; **F2**: Ruminococcaceae and Th17; **F3**: Ruminococcaceae and Treg/Th17; **G1**: Enterobacteriaceae and Treg; **G2**: Enterobacteriaceae and Th17; **G3**: Enterobacteriaceae and Treg/Th17).

Quantitative evaluation of circulating Treg and Th17 cells was performed at the engraftment time post-transplantation (Figures 6A–D). The aGVHD group harbored a fewer proportion of Treg cells compared with the non-aGVHD group [2.22% (range: 0.33–5.90%) versus 3.35% (range: 0.61–10.60%); *p* < 0.001] and a greater proportion of Th17 cells compared with the non-aGVHD group [3.32% (range: 0.58–8.90%) versus 2.67% (range: 0.39–7.10%); *p* = 0.003].

The results further demonstrate that the relative abundance of the Lachnospiraceae and Ruminococcaceae families positively correlated with Treg cell counts (*r* = 0.578 and 0.492; *p* < 0.001 and <0.001, respectively; Figures 6E1,F1). Although Lachnospiraceae and Ruminococcaceae did not correlate with Th17 cell counts (*r* = −0.190 and −0.209; *p* = 0.090 and 0.061, respectively; Figures 6E2,F2), they positively correlated with the ratio of Treg and Th17 cells (*r* = 0.469 and 0.419; *p* < 0.001 and <0.001, respectively; Figures 6E3,F3). In contrast, the relative abundance of Enterobacteriaceae did not correlate with Treg cell counts (*r* = −0.065; *p* = 0.567; Figure 6G1), whereas Enterobacteriaceae positively correlated with Th17 cell counts (*r* = 0.388; *p* < 0.001; Figure 6G2). Furthermore, Enterobacteriaceae also negatively correlated with the Treg/Th17 cell ratio (*r* = −0.277, *p* = 0.012; Figure 6G3). No significant correlation was found between other families’ microbiota and T cell subsets.

### Acetylated Histone Level in CD4+ T Cells Correlated With the Microbiota and the Treg/Th17 Cell Ratio

**Figure 7:**
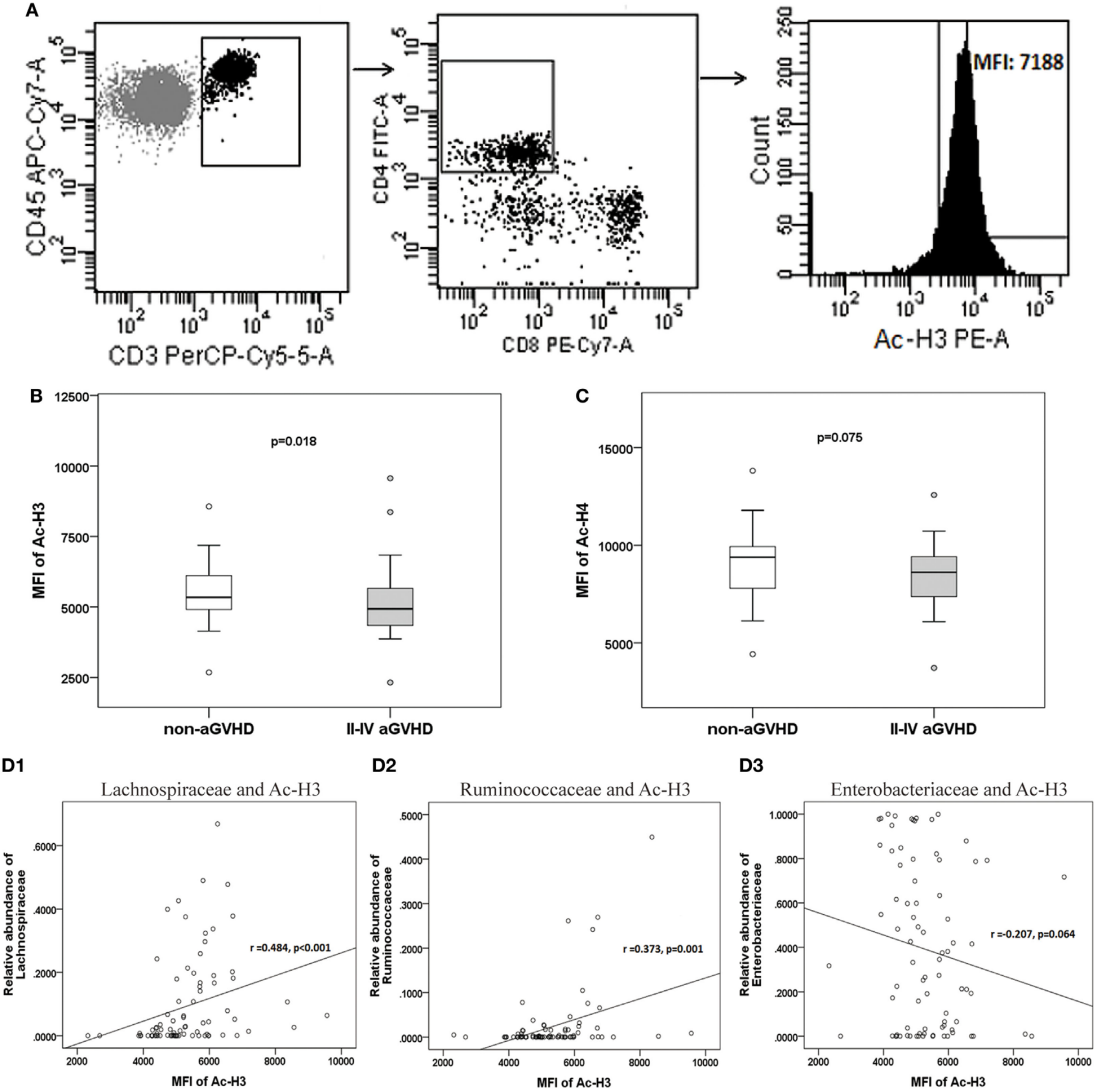
The level of acetylated histone is associated with acute graft-versus-host disease (aGVHD), and correlates with microbiota. **(A)** Peripheral mononuclear blood cells were examined by flow cytometry, after gating with CD45+ and CD3+, CD4+ and CD8−. The median fluorescence intensity (MFI) of the acetylated histones is shown in the histogram. **(B)** The MFIs for acetylated histone H3 (Ac-H3) and **(C)** Ac-H4 were compared between the aGVHD and non-aGVHD groups. The correlations between the relative abundance of the Lachnospiraceae **(D1)**, Ruminococcaceae **(D2)**, or Enterobacteriaceae families **(D3)**, and Ac-H3.

The expression of H3 and H4 acetylation in CD4+ CD8− T cell was detected, and the MFI of acetylated H3 and H4 was calculated (Figure 7A). We found that the levels of acetylated H3 were significantly lower in the aGVHD group (MFI: 4,934, range: 2,321–9,651) compared with the non-aGVHD group (5,342, range: 2,678–8,563; *p* = 0.018; Figure 7B). In contrast, the levels of acetylated H4 were not significantly different between the groups (8,617, range: 3,721–12,573 versus 9,389, range: 4,426–13,813; *p* = 0.075; Figure 7C).

**Figure 8:**
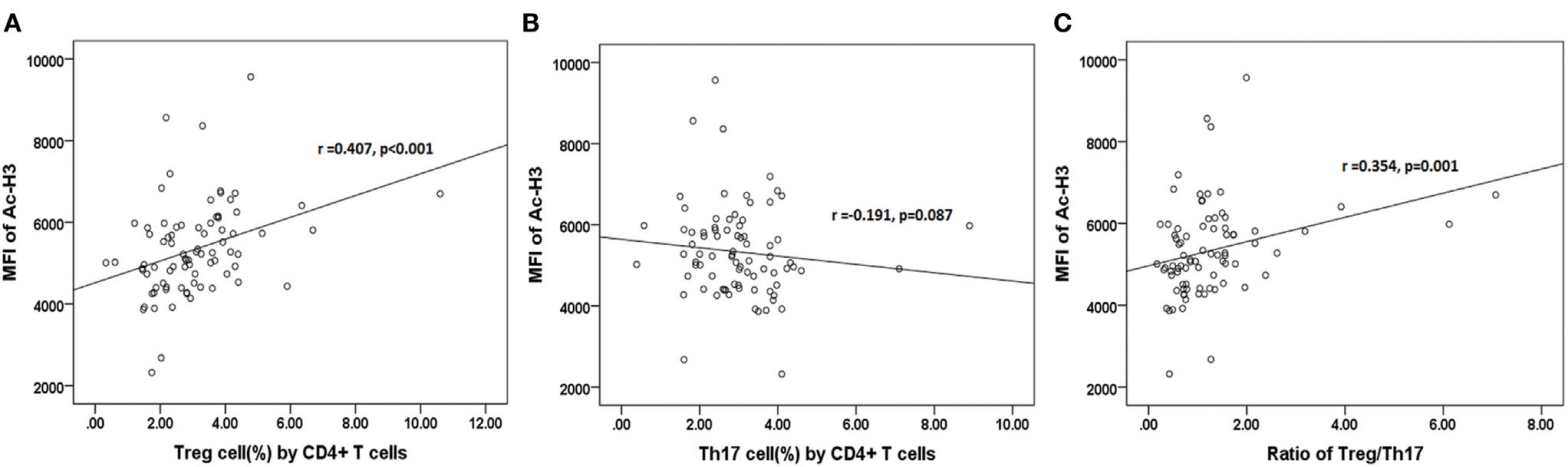
Correlations between acetylated histone H3 and percentages of Treg **(A)** or Th17 **(B)** cells, or the Treg/Th17 ratio **(C)**.

The further results revealed that the relative abundance of Lachnospiraceae and Ruminococcaceae positively correlated with H3 acetylation (*r* = 0.484 and 0.373; *p* < 0.001 and *p* = 0.001, respectively; Figures 7D1,D2), whereas the relative abundance of Enterobacteriaceae did not correlate with H3 acetylation (*r* = −0.207; *p* = 0.064; Figure 7D3). Furthermore, we found that the MFI of H3 acetylation positively correlated with Treg cell percentage (by CD4+ T cells) and the Treg/Th17 cell ratio (*r* = 0.407 and 0.354; *p* < 0.001 and *p* = 0.001, respectively; Figures 8A,C), although H3 acetylation did not directly correlate with Th17 cell percentage (*r* = −0.191; *p* = 0.087; Figure 8B). Additionally, the H4 acetylation level was not found to be correlated with the microbiota or T cell subsets (data not shown).

## Discussion

An increasing number of studies have demonstrated that microbiota composition is associated with the development of inflammatory bowel diseases, aGVHD, and even leukemia relapse in humans (7, 24, 33–35). In animal models, the mechanism of the effects of the microbiota on immune homeostasis has been widely studied (24, 33, 36). These studies have indicated that different microbiota drive or inhibit the activation of the immune system *via* their metabolites, such as SCFAs, histone deacetylase inhibitors, or lipopolysaccharide (LPS), a potent inflammatory activator (24, 37–39). Metabolites from Clostridia (e.g., Lachnospiraceae and Ruminococcaceae) induce immune tolerance through Tregs cells, whereas metabolites from Gammaproteobacteria (e.g., Enterobacteriaceae) activate immune responses through Th17 cells (17, 19, 24, 39). However, the mechanism underlying the effects of the microbiota on immune homeostasis in recipients undergoing allo-HSCT has not been determined. We observed that the intestinal microbiota is associated with the development of aGVHD. Furthermore, there were specific relationships between the different microbiota taxa and the balance of Treg and Th17 cells, and the relative abundance of the Lachnospiraceae and Ruminococcaceae families positively correlated with the Treg/Th17 cell ratio, and Enterobacteriaceae negatively correlated with the Treg/Th17 cell ratio. In addition, the level of H3 acetylation was associated with the relative abundance of Lachnospiraceae, Ruminococcaceae, and the balance of Treg and Th17 cells. We speculated that the intestinal microbiota might influence the development of aGVHD by coordinating the Treg/Th17 balance at engraftment post-transplantation, which might work through H3 acetylation in CD4 T cells.

Many factors influenced the intestinal microbiota during allo-HSCT, such as antibiotics, conditioning regimens, diet, and infections (7, 13, 40–42). A variety of studies have demonstrated that the early use of broad-spectrum antibiotics result in the loss of intestinal microbiota diversity and an increase in aGVHD during post-transplantation (13, 15, 43). Taur Y et al. (7) reported that low microbiota diversity was associated with antibiotics administration and myeloablative conditioning. Y. Shono observed that treatment with antibiotics with increased activity against anaerobes correlated with increased aGVHD-related mortality and altered intestinal microbiota in allo-HSCT patients with neutropenic fever (44). Simms-Waldrip et al. (13) reported that decreased Clostridia were associated with antibiotics. However, these studies did not indicate special associations with types or families of bacteria that are influenced by special antibiotics or conditioning regimens. In this study, we provide the first evidence that loss of Lachnospiraceae is associated with b-lactam antibiotic administration, and a bloom in Enterobacteriaceae is associated with vancomycin in addition to their effects on microbiota diversity. Interestingly, we found that loss of Lachnospiraceae and Ruminococcaceae is also associated with intensified conditioning. However, we did not find other factors, such as patient age, HCT-CI, donor type and source, TBI conditioning regimen, bloodstream infection, and amikacin administration, associated with microbiota. These findings could be interpreted as b-lactam antibiotics and intensified conditioning could result in higher levels of oxygen in the gut, which inhibits the propagation of anaerobic bacteria (i.e., Lachnospiraceae and Ruminococcaceae), while vancomycin kills coccus at a certain, resulting in a relative bloom in Enterobacteriaceae (24, 45). Alternatively, intestinal epithelium damage due to conditioning could also contribute to changes in microbiota composition *via* alterations in the expression of antimicrobial molecules produced by intestinal epithelial cells (41).

Commensal intestinal bacteria have long been implicated in the development of aGVHD (12, 46). In this study, because the intestinal microbiota was impacted by b-lactam antibiotics, it is not surprising that b-lactam antibiotics impact the development of aGVHD. According to Cox proportional hazards analysis for aGVHD, b-lactam antibiotics administration and low microbiota diversity are independent risk factors for aGVHD, which is consistent with previous studies (7). Although intensified conditioning was not an independent risk factor for aGVHD, Jagasia et al. (47) reported that not just conditioning intensity, but the intensity and TBI plus graft source have a combined effect on the risk for aGVHD, and the limited cases in this study might be another reason. Our results also indicated that vancomycin is not associated with aGVHD although the microbiota diversity was impacted by vancomycin. We speculated that vancomycin, in addition to its specificities, was likely to work to a lesser degree than broad-spectrum antibiotics against obligate anaerobic bacteria (i.e., Lachnospiraceae), and the association between vancomycin and aGVHD was confounded by other antibiotics (i.e., b-lactam antibiotics) that were frequently and concurrently given, which significantly altered the intestinal microbiota (7).

Furthermore, we observed that the decrease in anti-inflammatory Clostridia, particularly in the Lachnospiraceae family and the Blautia genus, and increase in proinflammatory bacteria (i.e., Enterobacteriaceae) were associated with aGVHD (13, 16). Additionally, other bacterials, such as the Ruminococcaceae and Peptostreptococcaceae families, and *Lachnoclostridium* genus, were newly found to be associated with aGVHD in humans, and these Clostridia have been shown to alleviate IBD and GVHD in mice (14, 20, 48). The finding of microbiota in aGVHD could also be interpreted as microbiota taxa are impacted by antibiotics administration, different conditioning regimens, diet, and individual variability.

In animal experiments, the mechanism of the effects of commensal microbiota on immune homeostasis has been studied extensively (14, 23, 24). On the one hand, some studies have demonstrated that anti-inflammatory Clostridia could coordinate the Treg/Th17 balance and induce tolerance by histone deacetylase inhibition (17, 19, 20, 23, 49). Metabolites of Clostridia, such as SCFAs (i.e., epigenetic modification of the Foxp3 promoter), promote histone acetylation and induce the differentiation of Treg cells (17, 20, 22, 23, 49). On the other hand, studies have indicated that metabolites from pathogenic Enterobacteriaceae, such as LPS, elicit a T cell response and promote Th17-mediated inflammation (24). In our study in humans, the results indicated that the abundance of Lachnospiraceae and Ruminococcaceae correlated with the acetylated H3 level in CD4+ T cells, and the level of acetylated H3 correlated with the Treg/Th17 balance. Furthermore, the results also demonstrated that the relative abundance of the Lachnospiraceae, Ruminococcaceae, and Enterobacteriaceae families correlated with the Treg/Th17 balance. We hypothesized that the intestinal microbiota might have an effect on immune homeostasis by coordinating the Treg/Th17 balance, which might be through the function of acetylated H3 in CD4 T cells during allo-HSCT. First, intestinal Clostridia-derived metabolites, SCFAs, play an important role in inducing the differentiation of regulatory T cells and modulating the Treg/Th17 balance by histone acetylation, particularly acetylated H3(17, 19, 20, 23, 49–52). Second, in many inflammatory conditions, such as conditioning injury and antibiotic treatment, the levels of oxygen were higher in the gut, which permits aerobic respiration by Enterobacteriaceae, while inhibiting the growth of obligate anaerobes Clostridia (e.g., Lachnospiraceae and Ruminococcaceae) (24, 38). Enterobacteriaceae was thought to be potent inflammatory PAMPs and be involved in LPS biosynthesis, resulting in Th17 activation and further Treg/Th17 imbalance, promoting inflammatory disease and aGVHD (24, 37–39).

This study has some limitations. On the one hand, our results are not from a large number of cases because there are not enough patients, in particular, the limited volunteers, to enroll in this study in our single center, it needs to determined whether the results could be generalizable to more populations. On the other hand, it is a pity for lack of the baseline samples prior to the conditioning to explain the changes after transplantation.

## Conclusion

This study suggests that alterations in the intestinal microbiota at engraftment might impact immune homeostasis *via* the Treg/Th17 balance and modulate the development of aGVHD post-transplantation in humans, which might work by regulating the level of H3 acetylation in CD4+ T cells. Conversely, microbial alterations may be alleviated by preventively replenishing Lachnospiraceae and Ruminococcaceae or preemptively restricting of Enterobacterieaceae during allo-HSCT. In the future, large samples and prospective studies are needed to determine which intestinal microbiota contributes to mitigating aGVHD and improving outcomes of allo-HSCT.

## Ethics Statement

This study was approved by the ethical committee of the Nanfang Hospital, Southern Medical University, China. After approval of the study by ethical committee, the consent of the participants was signed for usage of biological samples for research purpose.

## Author Contributions

QLiu, LH, and HJ designed and wrote the paper. ZF, FH, LX, and MD contributed to data acquisition. LZ performed data analysis and interpretation. XZ detected the flow cytometry of blood specimen. QLin and HZ extracted the DNA of feces specimen. All authors have read and approved the final manuscript and agreed to be accountable for the whole work.

## Conflict of Interest Statement

The authors declared that the study was conducted in the absence of any financial or commercial relationships that could be construed as potential conflicts of interest.
