# Supplementary material for: Intestinal Microbiota at Engraftment Influence Acute Graft-Versus-Host Disease via the Treg/Th17 Balance in Allo-HSCT Recipients
Source: Front Immunol. 2018 Apr 24;9:669. doi: 10.3389/fimmu.2018.00669 (PMC5928130; doi:10.3389/fimmu.2018.00669)
Supplement: Supplementary file 1 [file Presentation_1.PPT]

## Slide 1
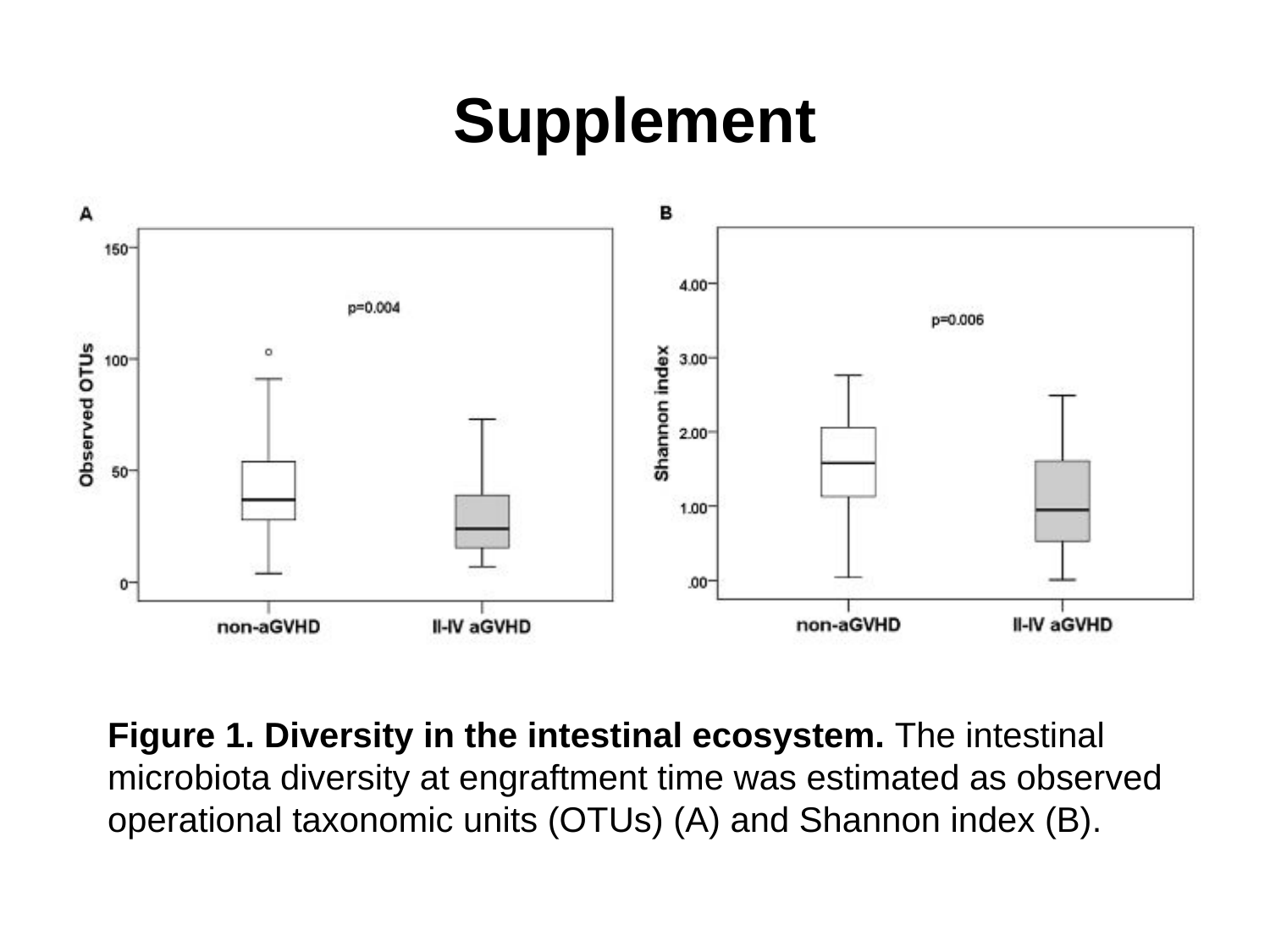

# Supplement
Figure 1. Diversity in the intestinal ecosystem. The intestinal microbiota diversity at engraftment time was estimated as observed operational taxonomic units (OTUs) (A) and Shannon index (B).
